# Targeting tumor-associated macrophages through phytochemicals: a promising strategy for cold tumor therapy

**DOI:** 10.1186/s13020-026-01401-4

**Published:** 2026-04-28

**Authors:** Tian Shue, Yujun Guo, Peishan Zhou, Dan Yao, Huan Liao, Chengxin Long, Yulin Qi, Luyao Yang, Nianzhi Chen

**Affiliations:** 1 Department of Dermatologyermatology, Deyang Hospital Affiliated Hospital of Chengdu University of Traditional Chinese Medicine, Deyang, China; 2 College of Biomedical Engineering, Chongqing Medical University, Chongqing, China; 3 Department of Medical Oncology & Cancer Institute of Integrative Medicine, Shuguang hospital, Shanghai University of Traditional Chinese Medicine, Shanghai, China; 4 Hospital of Chengdu University of Traditional Chinese Medicine, Chengdu, China; 5 Central Laboratory, Shenzhen Bao’an District Songgang People’s Hospital, Shenzhen, China

**Keywords:** Natural products, TAMs, Immunotherapy, Cold tumors

## Abstract

Immunotherapy serves as the fourth modality for cancer treatment, following surgery, radiotherapy, and chemotherapy. However, its efficacy remains inconsistent due to the heterogeneity of the tumor microenvironment (TME). Tumors characterized by an immunosuppressive TME that are unresponsive to immunotherapy are termed “cold tumors.” Thus, strategies to reverse this immunosuppressive environment are promising for treating cold tumors. Tumor-associated macrophages (TAMs) play a significant role in this process, making TAM-targeting therapies potentially valuable. Natural products (NPs), which are bioactive compounds from traditional herbs, exhibit broad anticancer effects and modulate TAM polarization, positioning them as a promising source for anticancer agents. This article explores the relationship between TAMs and the TME, emphasizing the mechanisms by which natural products, such as terpenoids and quinones, influence TAM polarization to inhibit tumor progression. By analyzing TAM roles in the TME and summarizing existing natural products targeting TAM polarization, this study offers insights for developing novel TAM-targeting drugs, and aids in the conversion of cold tumors to hot tumors.

**Graphical Abstract:**

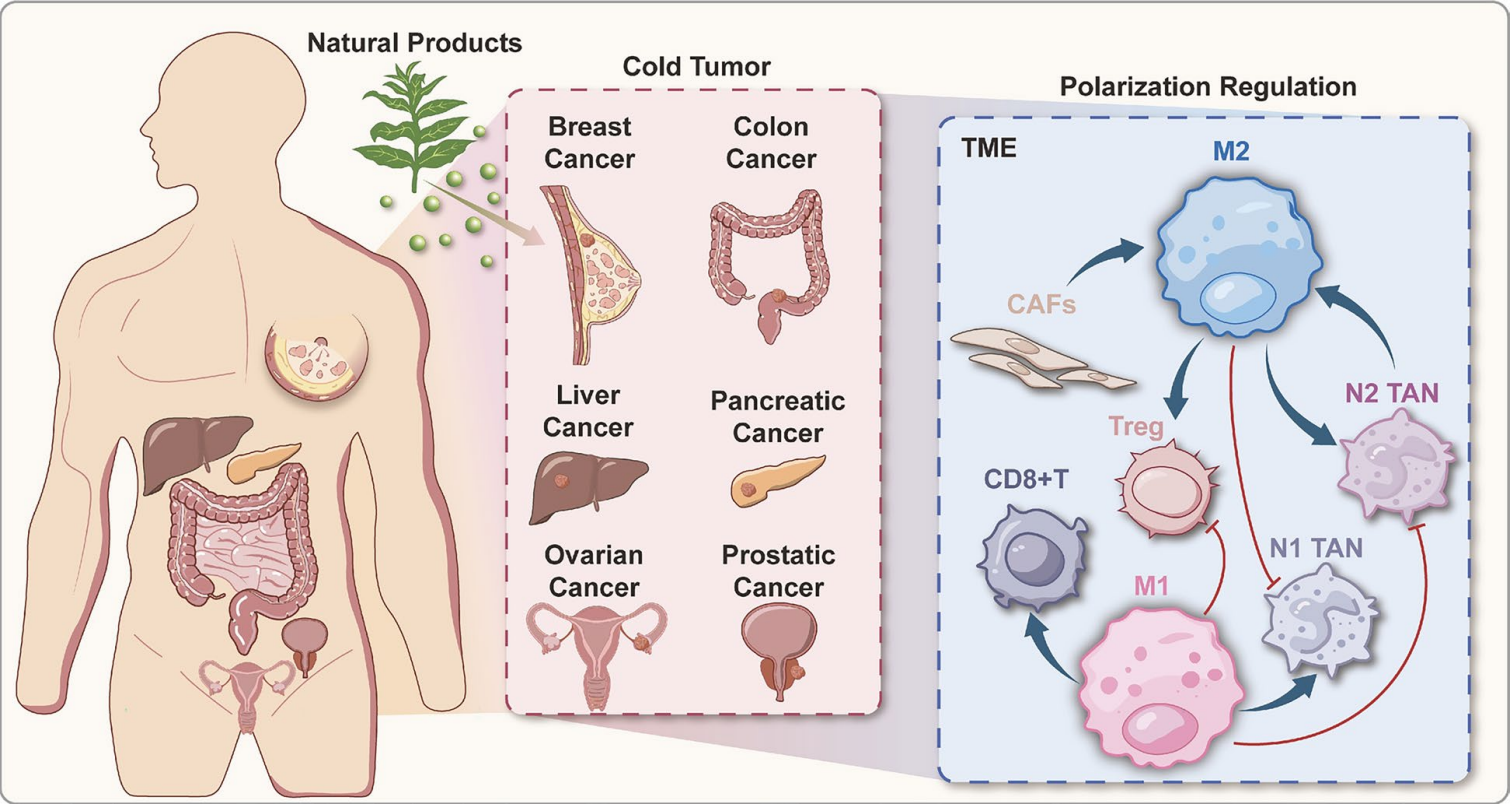

**Supplementary Information:**

The online version contains supplementary material available at 10.1186/s13020-026-01401-4.

## Highlights

- TAM polarization and its interactions with other cells shape the immunosuppressive state of the TME
- Targeting TAM polarization with natural products is a promising strategy to reverse TME immunosuppression
- Natural products targeting TAM polarization offer new directions for specific immunomodulator development

**Supplementary Information:**

The online version contains supplementary material available at 10.1186/s13020-026-01401-4.

## Introduction

Cancer is a leading cause of death globally [133, 135], Cancer treatment is shifting from direct cytotoxicity to immune modulation, with significant advancements in adoptive cell therapy (ACT) and immune checkpoint inhibitors (ICIs) that enhance tumor-specific immunity [27, 102], Tingrui [174, 175, 177], Some patients cannot benefit from immunotherapy due to an immunosuppressive tumor microenvironment (TME), marked by peripheral CD8 + T cells, low PD-L1 expression, and various immunosuppressive cells [60],Y. [126, 127, 160],These tumors, known as “cold tumors,” include prostate and pancreatic cancers with immunosuppressive TMEs, breast cancers with low T cell infiltration, and gliomas with abundant immunosuppressive macrophages, leading to reduced immunotherapy efficacy [8, 39],Y. [77, 80, 154],This highlights the need to reshape the TME immune ecology in cold tumors.

The TME is crucial in tumor development and immunotherapy due to its immune cells, including NK cells, macrophages, and T cells [1, 107, 178], Tumor-associated macrophages (TAMs) make up 50% of the tumor mass [97], Highly infiltrated TAMs are critical in tumor progression, metastasis, and immunotherapy, with their subpopulations directly influencing anti-tumor strategies [148]. TAMs are classified into M1 and M2 types, M1 macrophages secrete IL-1β, IL-6, and TNF-α, promoting anti-cancer effects, while M2 macrophages secrete TGF-β and IL-10, facilitating tumor progression and immune evasion. [34].Targeting TAM polarization is a key strategy to overcome immune suppression in cold tumors.

About 50% of anti-tumor drugs are derived from natural products, which are preferred over conventional chemotherapeutics due to their diverse bioactivity and low toxicity [101], Moreover, numerous studies have demonstrated that natural products regulate innate immune cells, including TAMs (H. [161, 163]). This review discusses the immune-suppressive environment resulting from the crosstalk between TAMs and other cells in the TME, summarizing the mechanisms by which natural products target TAM polarization in cold tumors. It aims to provide structural insights and targets for novel TAM-targeting drugs and new candidates for immunotherapy.

## Functional changes of TAMs in the TME

A fundamental understanding of TAM-mediated immunomodulation is a prerequisite for discussing the influence of natural products on these cells. In the TME, the dynamic changes in TAM phenotypes influence the direction of tumor immune responses. Circulating monocytes are recruited to become TAMs by chemokines such as CCL2 and CCL5 within the TME [149], Subsequently, macrophages differentiate into M1 (inflammatory/anti-tumor) or M2 (anti-inflammatory/pro-tumor) phenotypes, with M2 macrophages linked to cancer progression [5, 63, 103, 148], Understanding the induction of M1 and M2 phenotypes and their contribution to an immunosuppressive environment enables the development of targeted modulation strategies.

### M1 macrophages

TAMs express various receptors, including Toll-like receptors (TLRs), a subfamily of pattern recognition receptors that detect invading pathogens and endogenous harmful stimuli, thereby inducing innate and adaptive immune responses.

Among TLRs, TLR4 serves as the primary receptor for lipopolysaccharides (LPS). Upon LPS binding, the TLR4-MD2 complex recruits adaptor molecules with TIR domains, such as TIRAP (Mal) and MyD88 (MyD88-dependent pathway), to transmit early activation signals to NF-κB. This activation leads to the production of pro-inflammatory cytokines (e.g., TNF-α, IFN-γ), promoting M1 polarization of TAMs [57, 100, 114], therefore, researchers aim to enhance pro-inflammatory factor secretion using drugs to directly activate M1 macrophages via the TLR4-mediated NF-κB pathway [176, 189].

M1 macrophages secrete pro-inflammatory factors like IL-6, IL-12, IL-23, TNF-α, nitric oxide (NO), and reactive oxygen species (ROS). Cytokines such as IL-1β induce PD-L1 expression in liver cancer cells via transcription factors p65 and IRF1 [190], TNF-α significantly increases PD-L1 abundance through CDK4/UPS14 [147]. Therefore, promoting M1 macrophages within tumors enhances the efficacy of immunotherapy.

### M2 macrophages

M2 macrophages are typically activated by macrophage colony-stimulating factor (M-CSF), IL-13, TGF-β, and Th2 cells. Based on the stimuli received, M2 subtypes are classified into four types: M2a (activated by IL-4 and IL-13), M2b (activated by immune complexes, Toll-like receptor ligands, and IL-1β), M2c (activated by glucocorticoids, IL-10, and TGF-β), and M2d (activated by IL-6 and adenosine) [7, 18, 38, 78, 87]. Additionally, Tumor-derived substances can induce M2 macrophage polarization. Lactate, for instance, promotes TAM M2 polarization via the mTOR/Akt473/CCL17 pathway, enhancing CCL17 secretion and facilitating pituitary adenoma invasion [171]. Exosomes are implicated in cancer progression. Gastric cancer-derived exosomal miR-519a-3p activates the MAPK/ERK pathway by targeting DUSP2, promoting M2-like polarization of intrahepatic macrophages and facilitating liver metastasis [111].

M2-like macrophages promote angiogenesis by secreting factors like PDGF and IGF. They also release immunosuppressive substances that inhibit Th1 activity and enhance Th2 responses, reducing inflammation control and facilitating tumor growth, resistance, and angiogenesis [5], Shujing [138, 139, 141].

## Crosstalk between TAM and immune cells in the TME

**Fig. 1:**
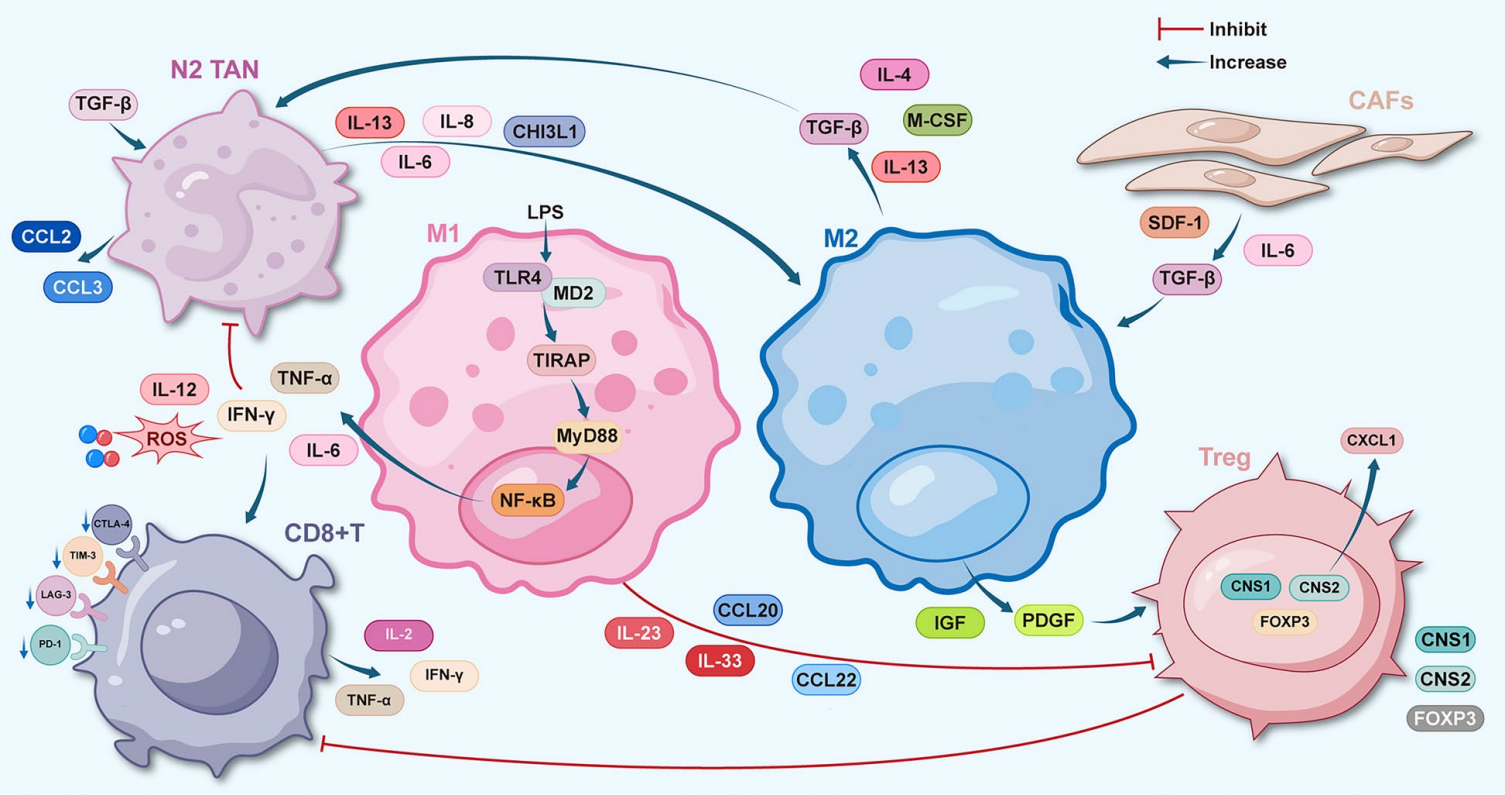
Macrophage and immune cell crosstalk. The mechanisms underlying the crosstalk between macrophages and various immune cells include: (1) The interaction of CD8^+^T cells with TAM antigen-specific synapses to induce T cell exhaustion; M2 polarization of macrophages suppresses CD8^+^T cell activity. (2) Factors such as IL-33, IL-23, and CCL20 in TAM can modulate Tregs, thereby fostering an immunosuppressive microenvironment. (3) Cytokines such as IL-2, IL-8, and CHI3L1 secreted by CAFs induce macrophage polarization, contributing to the establishment of an immunosuppressive milieu. (4) IL-13 secreted by TAM promotes M2 polarization of TAMs, consequently leading to immunosuppression

**Fig. 2:**
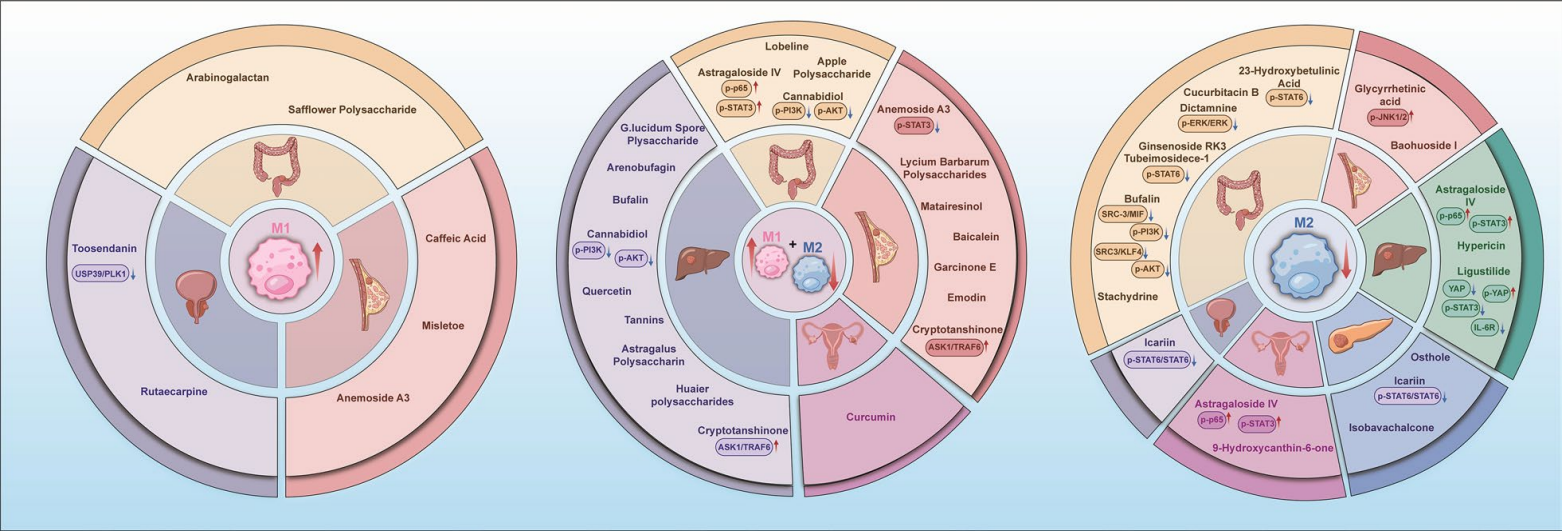
Summary of the effects of natural products on macrophage polarization. This figure illustrates the differential effects of various natural products on macrophage polarization. The left panel shows that specific natural products promote M1 polarization of TAMs in colorectal cancer (CRC), prostate cancer (PCa), and breast cancer (BC). The middle panel indicates that certain natural products enhance M1 polarization while simultaneously inhibiting M2 polarization in colorectal cancer (CRC), hepatocellular carcinoma (HCC), breast cancer (BC), and ovarian cancer (OC). The right panel shows that selected natural product inhibit M2 polarization of TAMs in colorectal cancer (CRC), breast cancer (BC), hepatocellular carcinoma (HCC), pancreatic ductal adenocarcinoma (PDAC), ovarian cancer (OC), and prostate cancer (PCa)

Beyond their intrinsic effects on the TME, TAMs typically engage in complex crosstalk with other microenvironmental components. TME comprises immune cells, cancer-associated fibroblasts (CAFs), and extracellular matrix (ECM) [41, 153], which interact to create an immunosuppressive environment (Fig. 2. T lymphocytes serve as the primary effector cells against cancer; however, tumor progression leads to T cell dysfunction and exhaustion [24], contributing to TME immunosuppression. Regulatory T cells (Tregs) inhibit CD4 + T cell activity, further enhancing this immunosuppressive milieu [117]. CAFs play a critical role in tumor progression by producing factors that influence other TME components, facilitating immune evasion [32]. Neutrophils and polymorphonuclear cells also significantly contribute to the TME, correlating with tumor proliferation and metastasis [6, 83, 148]. TAMs, as abundant immune cells, interact with T lymphocytes, Tregs, CAFs, and tumor-associated neutrophils (TANs), collectively fostering an immunosuppressive environment that promotes cancer development. [88], This section explores the crosstalk among these cells, offering insights for reversing tumor immunosuppression** (**Fig. 1**).**

### T lymphocytes and TAMs

T cells are essential for adaptive immunity, responding to antigens as CD4 + and CD8 + subsets. CD8 + T cells play a critical role in tumor regulation by secreting cytokines and cytotoxic factors to combat cancer. However, their function can decline or become exhausted, marked by increased expression of inhibitory receptors (PD-1, CTLA-4, TIM-3, LAG-3) and reduced cytokine production (IL-2, TNF, IFN-γ) [125, 130, 137], [59]. demonstrated that specific antigen-specific synapses between CD8 + T cells and TAMs contribute to T cell exhaustion [59], In breast cancer, arginine from tumor cells induces M2 polarization of macrophages via the TDG-PPARG axis, inhibiting CD8 + T cell activity. [188].

T cells are also classified into cytotoxic, helper, and regulatory T cells (Tregs), with Tregs characterized by CD4 + CD25 + [20], expression and FOXP3 [55], TAMs recruit Tregs through CCL20/CCL22, enhancing immunosuppression [132], For instance, TAMs secrete IL-23 to stabilize Tregs and CXCL1 to promote immune evasion in breast cancer (J. [64, 65, 67, 146]), In TAMs, upregulated IL-33 expression enhances Treg differentiation, contributing to the immunosuppressive microenvironment in lung cancer [52]. In hepatocellular carcinoma, galectin-1 (Gal1) activates the PI3K/AKT/NF-κB pathway, upregulating CCL20 and recruiting CCR6 + FOXP3 + Tregs, leading to CD8 + T cell dysfunction (Y. [138, 139, 141, 150, 167]) STAT3 is pivotal in regulating anti-tumor immunity [191], with targeting STAT3 disrupting Treg-M2 macrophage interactions and enhancing anti-tumor responses in colorectal cancer [46]. Thus, TAMs and T lymphocytes collaboratively shape the tumor immunosuppressive microenvironment.

### CAFs and TAMs

Fibroblasts maintain homeostasis, while tumor-associated fibroblasts (CAFs) promote angiogenesis and secrete growth factors, influencing immune balance in tumors [54, 89, 144]. Activated CAFs exhibit a dual role, [158, 159], but this discussion focuses on their function in promoting tumor growth and suppressing immunity.

CAFs exert their functions within the TME through autocrine and paracrine mechanisms. The cytokines they secrete engage in transmembrane signaling with surrounding cells to recruit immune cells to the tumor site; however, the recruited immune cells contribute to the formation of an immunosuppressive microenvironment. [48, 172, 182]. CAFs promote immunosuppression by producing cytokines like TGF-β, IL-6, and SDF-1, while also secreting IL-2, IL-10, IL-8, CCL2 and CHI3L1 to induce macrophage polarization, enhancing TAM tumor-promoting capabilities (S. [12, 14, 62],Chao [64, 65, 67],Q. [48]) CAF-derived IGFBP7 induces M2 polarization in gliomas, and the FGF2/FGFR1/PI3K/AKT pathway promotes M2 polarization in gastric cancer [66],X. [174, 175, 177], Additionally, macrophages acquire tumor-promoting properties through the CAF-driven CXCL12-CXCR4 axis, supporting an immunosuppressive microenvironment [129], thereby shaping an immunosuppressive microenvironment.

### TAN and TAM

Neutrophils are essential for innate immunity against pathogens. Granulocyte colony-stimulating factor (G-CSF) polarizes neutrophils to the anti-tumor (N1) phenotype, while transforming growth factor β (TGF-β) from tumors induces the pro-tumor (N2) phenotype. N1 tumor-associated neutrophils (TAN) exhibit high levels of TNFα, CCL3, and ICAM-1, with low arginase levels, while N2 neutrophils show increased chemokines like CCL2 and CCL3 [33, 43, 91, 149].

The tumor microenvironment promotes TAMs and TANs to adopt a pro-tumor phenotype, aiding tumor growth and invasion. Neutrophils recruit macrophages, which subsequently affect neutrophil function [112], For example, IL-13 secreted by neutrophils promotes M2 polarization of TAMs [58], Co-culturing TANs and TAMs results in higher levels of oncostatin M (a cytokine from the IL-6 family) and IL-11, both of which activate STAT3 signaling in intrahepatic cholangiocarcinoma cells, further promoting tumor progression [186]. In liver cancer, neutrophil-derived CCL2 recruits macrophages, promoting angiogenesis, enhancing tumor growth and metastasis, and causing sorafenib resistance [185]. Thus, the interaction between TANs and TAMs collectively contributes to tumor progression and development.

## The natural products targeting macrophage polarization

In summary, the polarization phenotypes of TAMs and their intricate crosstalk with other immune cells within the TME establish a robust immunosuppressive barrier. This mechanism serves as a pivotal determinant of the insensitivity of "cold tumors" to immunotherapy. Nevertheless, this suppressive state is not irreversible. Given the critical role of macrophage polarization in the treatment of cold tumors, targeting macrophage polarization presents significant therapeutic potential. The use of natural products has a long history, serving as inspiration for drug design [22], recently, natural products and their derivatives have captured a substantial market share [15], demonstrating their efficacy in oncology. Most natural products exhibit anticancer properties, The polarization effects of different natural products on macrophages in various cold tumors can be broadly categorized into three types **(**Fig. 2**)** The mechanisms underlying their induction of polarization will be detailed in the following sections (Table 1). The structures of all compounds are shown in the figure S1.

**Table 1:** Mechanisms of regulating cold tumors by different compounds

Name	Source	Cancer	Modeling (in vitro)	Modeling(in vivo)	Dose	Mechanism	References
Terpenoid							
Toosendanin	Melia toosendan	prostate cancer	DU145 LNCaP WPMY1 THP-1	BALB/c xenograft models	20 nM(in vitro) 0.2 mg/kg(in vivo)	CD86↑ USP39/PLK1↓	[122]
Glycyrrhetinic acid	Licorice	Breast cancer	RAW264.7 THP-1 4T1 MDA-MB-231	4T1 xenograft mouse model	10, 20, 40 μM(in vitro) 50, 100 mg/kg(in vivo)	CD206、ARG1、IL-10↓ p-JNK1/2↑	[19]
23-Hydroxybetulinic Acid	Betula platyphylla Sukaczev	colorectal cancer	THP-1 SW480 CT26	Allograft model	10, 20 μM(in vitro) 7.5, 15 mg/kg(in vivo)	p-STAT6、CD206↓	[31]
Cucurbitacin B	Cucurbitaceae	colorectal cancer	RAW264.7 THP-1 HCT116 CT26	AOM /DSS induced CRC model	400, 800 nM(in vitro) 0.5, 1 mg/kg(in vivo)	p-STAT3、p-JAK2↓ YM-1、Fizz-1、IL-10、ARG1↓ CD206↓	[181]
Quinones							
Emodin	Rheum	Breast cancer	E0771 4T1 Peritoneal macrophages	orthotopic breast tumor	10, 20, 50 μM 40 mg/kg(in vivo)	ARG1,CD206↓ iNOS↑ IRF4、STAT6、 C/EBPb↓	[49]
Cryptotanshinone	Salvia miltiorrhiza	Breast cancer	MDA-MB-231 RAW 264.7 BMDM	Orthotopic breast tumor	5, 10, 20 μM(in vitro) NA(in vivo)	iNOS↑MR↓ CD80, CD86↑CD206↓ TNF-α, IL-6,IL-12↑IL-10↓ ASK1/TRAF6↑	[164]
Cryptotanshinone	Salvia miltiorrhiza	Liver cancer	Hepa1-6 H22 Peritoneal Primary Macrophages	Subcutaneous tumor Mice	10 μM(in vitro) 40 mg/kg(in vivo)	CD86、M1↑ CD206、M2↓ p-p65、p-IκBα、HIF-1α↓ p-AMPK↑	[53]
Ligustilide	Radix Angelica Sinensis	Liver cancer	HepG2 THP-1	NA	100 μM(in vitro)	CD163、CD206、CCL22、ARG1、 TGF-β、IL-10↓ YAP↓p-YAP↑ IL-6R、p-STAT3↓	[162]
Hypericin	Hypericum perforatum	Liver cancer	Hepa1–6 H22 BMDM	subcutaneous tumor model	10, 20, 40 μmol/ml(in vitro) 10, 20 mg/kg(in vivo)	ARG1、Fizz1、YM1、IRF4、CD206、PPARγ、CCL24、IL-10、M2↓	[145]
Flavonoids							
Isobavachalcone	Psoralea corylifolia L	Pancreatic cancer	RAW264.7; Panc02 MDSCs	Suppressed Orthotopic Pancreatic Cancer in Mice	10、20 μM in vitro); 20 mg/kg(in vivo)	CD8 + T↑; Arg1、MRC1、TGF-β↓; M2↓	[79]
Icariin	Herba Epimedii	Pancreatic cancer	Panc02 RAW264.7	orthotopic pancreatic tumor progression in mice	20 μM, 80 μM(in vitro) ; 120 mg/kg(in vivo)	M2、CD206↓ MRC1、Arg1↓ p-STAT6/STAT6↓	[183]
Icariin	Herba Epimedii	prostate cancer	RM1 RAW264.7 BMDM	prostate cancer bone metastasis	30 μM(in vitro) 20 mg/kg, 40 mg/kg(in vivo)	CD206↓ CCL5、SPI1↓ CTSK、RANKL↓	[11]
garcinone E	Mangosteen	Breast cancer	RAW 264.7 THP-1 MDA-MB-231 MCF-7 HUVECs	tail vein breast cancer metastasis model	25, 50, 100 nM(in vitro) 2 mg/kg(in vivo)	CD206 、ARG1, Fizz1、Ym1↓ iNOS、CD86↑ p-STAT6↓	[94]
Baohuoside I	Epimedium koreanum Nakai	Breast cancer	4T1 RAW 264.7 THP-1	orthotopic breast tumor	1.25, 2.5, 5 μM(in vitro) 20 mg/kg(in vivo)	CD206↓、CXCL1↓	[138, 139, 141]
Baicalein	Scutellaria baicalensis Georgi	Breast cancer	MCF-7 MDA-MB-231 THP-1	Allograft transplantation model	20, 40 μM(in vitro) 50 mg/kg(in vivo)	CD206、ARG1、TGF-β1、IL-10↓, CD86、IL-12, TNF-α↑	180
Quercetin	tea and onion	Liver cancer	H22 HepG2	Homologous Tumor Transplantation	25, 50、100 mg/kg(in vivo)	CD86↑、CD206、PD-L1、TNF-α↓	[151]
Coumarins							
Osthole	Cnidium monnieri (Fructus Cnidii)	Pancreatic cancer	Panc02 RAW264.7 BMDM	attenuates orthotopic pancreatic tumors in mice	20 μM, 80 μM(in vitro) 120 mg/kg(in vivio)	M2、CD206↓ p-STAT6-p-ERK1/2-C/EBP β↓ MRC1、Ccl22、TGF-β↓	[133, 135]
Lignans							
Matairesinol	Podocarpus macrophyllus (Thunb.) Sweet	Breast cancer	THP-1 MDA-MB-231	NA	0.25, 0.5, 1ug/ml(in vitro)	CD86、CD16、CCR7、TNF-α↑ CD206、IL-10、TGF-β1、TGM2、MARCO↓	[10]
Phenolic acids							
Caffeic acid	Plants、fruit and vegetables	Breast cancer	MDA-MB-231 MDA-MB-468	xenografts mouse model	100, 200, 300 μM(in vitro) 5, 15 mg/kg(in vivo)	CD80↑	[155]
Curcumin	Gingeraceae and Araceae	Ovarian cancer	SKOV3 OVCAR-3 TAMs	NA	5, 10, 20 μM	IL-10、TGF-β、CCL-23、CXCR2↓ IL-1、IL-12、TNF-α↑	Xi [70, 71]
Tannins	Phyllanthus emblica L	Liver cancer	H22, RAW264.7, BMDM Hepa1 –6	Subcutaneous H22 tumor-bearing Mice Orthotopic Hepa1–6 tumor model	5, 10, 20 μg/ml(in vitro) 2, 1 g/kg(in vivo)	IL-6、TNF-α、IL12p40、iNOS↑ IL-10、ARG1、CD206、TGF-β↓	[9]
Alkaloids							
9-Hydroxycanthin-6-one	Ailanthus altissima	Ovarian cancer	A2780, SKOV3 OVCAR3 THP-1	NA	10, 20, 40 μM(in vitro)	MR, Trem2、IL-10↓	[50]
Stachydrine	Leonurus artemisia	colorectal cancer	MC38 HUVECs BMDMs	liver metastasis model	15, 30, 60 mg/kg(in vivo)	CD206、 ARG1、Fizz1、Mgl2、TGF-β1↓CD11c↑	[37]
Lobeline	Lobelia inflata	colorectal cancer	MC38 CT26 293 T	MC38 Xenograft model	30 μM(in vitro) 12.5, 25, 50 mg/kg(in vivo)	CD86、CD80、IL-1b、Tnf、Cxcl9、IL12a、Ccr7、Cxcl9↑ CD206、Tgfβ1、Vegfα、Egf、IL6、IL10、Arg1、Ccl22↓ IFN-γ-JAK-STAT1↑ IL-6-JAK-STAT3↓ MAPK14/p53/Slurp1	[179]
dictamnine	Dictamnus dasycarpus Turcz	colorectal cancer	DLD-1 LoVo THP-1	Xenograft tumor model	25, 50, 100 μM(in vitro) 50 mg/kg(in vivo)	ARG1、TGF-β、IL-10、CD163↓ p-ERK/ERK↓	[192]
Rutaecarpine	Euodia ruticarpa	Prostate cancer	TRAMP-C1	Ectopic mouse tumor models	7, 35, 70 mg/kg(in vivo)	TNF-α、IL-1β、 IL-6↑ CD4 + 、CD8 + 、CD19 + ↑	[75]
Polysaccharide							
Lycium barbarum polysaccharides	Lycii fructus	Breast cancer	4T1 BMDMs	orthotopic breast tumor	< 3KDa 3-50KDa 50-100KDa > 100KDa (in vitro) 50-100KDa(in vivo)	CD206、ARG1、Chil3↓ CD86、NO、IL-1β、TNF-α↑ TLR4MyD88-IKK-NF-κB↑	[76]
Apple polysaccharide	Apple	colorectal cancer	Raw264.7 colonic macrophages	AOM /DSS induced CRC model	0.5 mg/ml(in vitro) 5%(in vivo)	M1、iNOS、TNF-α、IL-23↑ ARG1、IL-4、IL-10↓ TLR4↑	[124]
Safflower Polysaccharide	Carthamus tinctorius L	colorectal cancer	HCT116, RKO, SW480, LoVo, MC38, RAW 264.7	AOM /DSS induced CRC model	100、500、1000 μg/ml(in vitro) 50 mg/kg(in vivo)	iNOS、IL-23↑ CD80、CD16↑ p-p65、p-IκBα↑ TNF-α、IL-6、NO↑	Q. [136, 140]
Arabinogalactan	Pollen Typhae	colorectal cancer	RAW264.7 RKO	NA	100, 200, 400 μg/ml	p-p65、p-IκBα↑ iNOS、TNF-α↑ CD86、CD16↑	[158, 159]
Astragalus polysaccharin	Astragalus propinquus Schischk	Liver cancer	MHCC97H Huh7 THP-1	Subcutaneous tumor Mice	8, 16 mg/ml(in vitro) 50, 100, 200 mg/kg(in vivo)	IL-10、ARG1、CD206、M2、CD68↓ iNOS、TNF-α、IL-1β、M1、CD86↑	[64, 65, 67]
Huaier polysaccharides	Trametes robiniophila Murr	Liver cancer	Hepa1–6 Huh-7 RAW264.7 THP-1	Xenograft models	0.5, 1, 2 mg/ml(in vitro) 4、8 g/kg(in vivo)	CD206↓ TNF-α、IL-9、IL-1β↑	[70, 71]
G. lucidum spore polysaccharide	Ganoderma lucidum	Liver cancer	H22 Peritoneal macrophages	NA	200, 400, 800 μg/ml(in vitro)	M1、CD86、TNF-α、IL-1β、IL-6、TGF-β1↑ CD206↓ PI3K、p-AKT↓BAX↑BCL2↓、Caspase9↑	[121]
Saponins							
Anemoside A3	Pulsatilla chinensis	Breast cancer	L929 4T1 BMDM	tail vein breast cancer metastasis model	50, 100ug/ml (in vitro) 5, 10, 20 mg/kg (in vivo)	CD86、IL-6、IL-1β↑ CD206, Arg1, Fizz1、Ym1↓ p-STAT3↓	P. [77, 80]
Anemoside A3	Pulsatilla chinensis	Breast cancer	THP-1 4T1 MCF-7	Orthotopic breast cancer model	50, 100 μg/ml 5, 10, 20 mg/kg (in vivo)	CD86, TNF-α、IL-12↑ IL-6, IL-1β↑ TLR4↑	[165]
Astragaloside IV	Radix Astragali	Ovarian cancer	A2780 SKOV3 THP-1	NA	0.5, 1, 5, 10 μg/ml	CD206, ARG1, IL-10, CCL24, PPARγ↓ TLR4↓	X. [136, 140])
Astragalcoside IV	Astragalus membranaceus	colorectal cancer	MC38 GW4869 RAW264.7	CRC liver metastasis model	12.5、25、50 μM (in vitro) 50 mg/ml (in vivo)	M1、CD86↑ M2、CD206↓	[184]
Astragaloside IV	Radix Astragali	Liver cancer	Huh-7 THP-1	Subcutaneous tumor Mice	50, 80, 100, 120, 150 μM 20, 40, 80, 100 mg/kg (in vivo)	M2、CD209、TGF-β↓ TLR4、p-p65、p-STAT3↑	[68]
Ginsenoside RK3	Panax ginseng	Glioblastoma	LN229, GL261 THP-1	glioma model	50, 100 μM (in vitro) 0.5, 1 mM (in vivo)	CD206、CD163、ARG1↓ PPARG、CCL2↓	[172, 182]
Tubeimosidce-1	Bolbostemmatis Rhizoma	colorectal cancer	MC38 CT26 RAW264.7	syngeneic subcutaneous CRC model	5, 10 μM 4 mg/kg(in vivo)	M2↓、CD8 + T↑ ARG1、IL-10、p-STAT6↓	D. [44, 45]
Steroids							
Bufalin	Bufo gargarizans	colorectal cancer	HCT116, HCT8 CT26 THP-1	xenograft model	0. 1, 0.5 mg/kg(in vivo)	CD68, CD206、IL-10,TGF-β↓ SRC-3/MIF↓	J. [12, 14]
Bufalin	Bufo gargarizans	colorectal cancer	HCT116 CT26 THP-1	syngeneic subcutaneous CRC model,	1 mg/kg(in vivo)	CD206、IL-10、TGF-β↓ SRC3/KLF4↓	[81]
Bufalin	Bufo gargarizans	colorectal cancer	CT26 THP-1	Xenograft Model	0.5 mg/kg(in vivo)	IL-10、TGF-β↓ CD11b + CD206 + ↓ HIF-1α、SRC-3↓	[134]
Bufalin	Bufo gargarizans	colorectal cancer	SW480, HCT8, LOVO, CACO2, HCT116, HT29, MC38, CT26	Spleen injection liver metastasis model	6, 12.5 nM(in Vitro) 0.5 mg/kg	CD206、TGF-β、IL-10、IL-6↓	D. [126, 127]
Bufalin	Bufo gargarizans	Liver cancer	Hepa1-6 BMDM	orthotopic HCC model	10 μg/kg(in vivo)	IL-23、IL-12、TNF-α、IFNγ、CD86、Ccr7、M1↑ IL-4、IL-13、IL-10、TGF-β、CD206、M2↓ P50 NFκB↓	[169]
Arenobufagin	Bufo gargarizans	Liver cancer	Hepa1–6 mouse peritoneal macrophages	transplanted tumor model	60 nM(in vitro) 1, 3, 6 mg/ml(in vivo)	CD86、TNF-α、ARG1、IL-6、IL-10、M1↑、CD206、M2↓ PCSK9↓LDLR↑	[72]
Others							
Cannabidiol	Cannabis sativa	colorectal cancer	MC38 BMDMs	syngeneic subcutaneous CRC model,	2.5, 5, 10 μM(in vitro) 5, 10 mg/kg(in vivo)	ARG1、CD206↓ iNOS↑ p-PI3K, p-Akt↓	[123]
Mistletoe	Viscum album L. var. coloratum	Breast cancer	MCF-7 THP-1	NA	100 pg/ml, 1 μg/ml(in vitro)	IL-6、TNF-α↑ IL-10、CCL18↓	[42]

### Terpenoid natural product

Approximately 60% of natural products are terpenes [23], which are natural compounds composed of isoprene units and are widely found in plants and animals. They exhibit diverse structures and physiological activities. Terpenes are classified based on the number of isoprene units, including monoterpenes, sesquiterpenes, and polyterpenes. In the process of remodeling the TME, the cornerstone of converting “cold” tumors into “hot” ones lies in abrogating immune tolerance and reinstating pro-inflammatory signaling cascades. Structurally diverse terpenoids primarily function by mimicking ligands to activate critical receptors, such as Toll-like receptors (TLRs), on the macrophage surface. By modulating JAK/STAT or NF-κB signaling pathways, these specific constituents induce the phenotypic repolarization of TAMs from an M2 to an M1 profile. This transition not only augments the secretion of pro-inflammatory cytokines, including TNF-α and IL-12, but also significantly enhances T-cell infiltration and activation within the tumor tissue. Consequently, this fundamentally transforms the “immune desert” into a pro-inflammatory milieu. The specific regulatory mechanisms of several terpenoid classes are delineated below.

Toosendanin (TSN) is a tetracyclic triterpenoid compound derived from *Melia toosendan*, known for its insecticidal [44, 45] and anticancer properties [51]. In prostate cancer, treatment with 20 nM TSN significantly increases the proportion of CD86 in THP-1 cells, promoting M1 polarization and exerting anticancer effects. This impact may be associated with ubiquitin-specific protease 39 (USP39) and polo-like kinase 1 (PLK1).

Glycyrrhetinic acid (GA) is a pentacyclic triterpenoid compound derived from *licorice,* known for its roles in regulating autophagic flux [30], anti-inflammatory, and antiviral effects, and it shows potential as a drug development scaffold [47]. In studies on breast cancer, 40 μM GA was found to be cytotoxic. In an M1 macrophage induction model (LPS + IFNγ), there was no significant difference in CD86 levels between the model group and the GA-treated group, However, in the M2 polarization model (IL-4 + IL-13), CD206 levels decreased compared to the model group. Additionally, mRNA expression analysis revealed no significant changes in M1 markers iNOS and TNF-α, while M2 markers Arg1 and CD206 were suppressed. Thus, GA inhibits M2 expression, potentially involving the JNK pathway [19].

23-Hydroxybetulinic Acid (23-HBA) is a pentacyclic triterpenoid derived from *Betula platyphylla Sukaczev,* known for its antitumor properties [187], In colorectal cancer, treatment with 20 μM 23-HBA inhibits the expression of CD206 in IL-10-induced M2 macrophages, potentially involving the STAT6 pathway [31].

Similarly, in colorectal cancer, Cucurbitacin B (CuB), a tetracyclic triterpenoid derived from the *Cucurbitaceae* family, exhibits comparable effects. CuB possesses anti-inflammatory, antitumor, and neuroprotective properties [21]. Treatment with 400 and 800 nM CuB in M2-polarized macrophages reduces the proportion of CD206, while also decreasing the mRNA expression of Ym-1, Fizz-1, IL-10, and Arg-1. The JAK2/STAT3 pathway is a key signaling pathway for M2 expression, and CuB can influence M2 polarization by modulating the JAK2/STAT3 pathway.

Thus, in existing studies on terpenoid natural products, TSN potentially drives M1 polarization by modulating the ubiquitination-associated USP39/PLK1 axis, thereby bolstering immune-mediated cytotoxicity. Conversely, the other three constituents primarily focus on suppressing M2 polarization: GA utilizes the JNK pathway to inhibit pro-tumorigenic phenotypes, while 23-HBA and CuB target the STAT6 and canonical JAK2/STAT3 axes, respectively. By intercepting these pivotal signal transductions, these compounds collectively facilitate a profound transition of the TME from a pro-tumorigenic to an anti-tumorigenic state.

### Quinones

Beyond the direct intervention of signaling pathways, reprogramming macrophage metabolism represents a pivotal strategy for orchestrating the transition from immunologically ‘cold’ to ‘hot’ tumors. Quinones are a class of organic compounds characterized by the presence of a cyclohexadiene-dione or cyclohexadiene-dimethylene structure. Based on their structure, they can be classified into categories such as benzoquinones, naphthoquinones, and anthraquinones. Quinones exhibit a wide range of biological activities, including antidiarrheal, anti-inflammatory, and antitumor effects [166]. Quinone compounds have been shown to be effective against various drug-resistant bacteria [26]. Concurrently, quinone exhibit potent metabolic regulatory capacity. By effectively inhibiting the oxidative phosphorylation upon which M2 macrophages depend, these compounds induce a metabolic shift toward glycolysis, thereby supporting an M1-like phenotype. This phenotypic remodeling enables TAMs to deplete immunosuppressive factors—such as TGF-β and IL-10—within the microenvironment and augment ROS production, effectively alleviating the suppression of cytotoxic T cells. Such a 'metabolic reprogramming' strategy provides a novel pharmacological rationale for revitalizing the immune activity of the TME.

Emodin (EM) is an anthraquinone compound derived from *Rheum*, characterized by the presence of an anthraquinone ring. It exhibits a variety of pharmacological effects and has been demonstrated to possess antibacterial, anti-allergic, and anticancer properties [28, 115]. In breast cancer, emodin (EM) treatment in peritoneal macrophages significantly increased the M1 marker iNOS. Additionally, levels of IRF4, STAT6, and C/EBPb, which regulate M2 polarization, decreased. Further analysis indicated that EM inhibits IRF4, CEBPb, and Arg1 by enhancing H3K27me3 levels in their promoter regions, thus epigenetically regulating macrophage polarization [49].

Cryptotanshinone (CPT) is an anthraquinone compound from *Salvia miltiorrhiza*. In breast cancer, CPT treatment increased CD80 and CD86 expression while decreasing CD206. This was accompanied by elevated levels of TNF-α, IL-6, and IL-12, and reduced IL-10, indicating that CPT inhibits tumor growth by suppressing M2 polarization and promoting M1 polarization. CPT's effects are linked to the cooperative action of ASK1 and TRAF6, which reprogram M2 macrophages and TAM to an M1 state [164]. In addition to its effects in breast cancer, the combination of Arsenic Trioxide and CPT (ACCS) in liver cancer also promotes M1 polarization and inhibits M2 polarization, activating the AMK pathway. [53]. CPT not only serves as an anticancer agent but also has potential as an immunomodulator.

Ligustilide (LIG) is a phenolic compound derived from *Radix Angelica Sinensis*, known for its various biological activities, including antispasmodic effects, renal protection, and anticancer properties [84, 156], Additionally, LIG can activate PINK1/Parkin-dependent mitophagy to alleviate neuronal damage following cerebral ischemi reduction in nuclear translocation, protein expression [90], and the transcriptional regulatory activity of YAP, along with an increase in p-YAP levels, thereby suppressing the activation of Yes-associated protein (YAP) [162].

Hypericin (Hyp) is an anthrone compound derived from *Hypericum perforatum*. Anthrones belong to the anthraquinone family, which can be reduced in acidic environments to form anthracenes and their tautomeric forms—anthrones. In liver cancer, studies using Hyp in the culture medium of IL-4-induced M2-type BMDM have shown that it can affect the proliferation, migration, and invasion of liver cancer cells. Additionally, Hyp significantly inhibits markers such as Arg1 and CD206, and its effects are associated with the PI3K/AKT signaling pathway [145].

This cohort of compounds demonstrates a profound mechanism of molecular intervention, spanning a cascade of responses from epigenetic landscapes to canonical intracellular kinase pathways. Specifically, EM leverages epigenetic modifications, utilizing H3K27me3 histone methylation to silence the expression of M2-associated genes. In contrast, CPT orchestrates macrophage ‘reprogramming’ via the ASK1/TRAF6 complex or the AMPK signaling pathway, effectively reverting TAMs from an M2-polarized state to an M1 phenotype. Concurrently, LIG targets the Hippo/YAP axis, sequestering YAP in the cytoplasm to attenuate M2-type transcriptional programs. Finally, Hyp targets the canonical PI3K/AKT pathway, leading to a significant downregulation of M2 markers. Collectively, these natural products disrupt the immunosuppressive state within the TME through multidimensional pathway intervention."

### Polyphenols

To convert immunologically “cold” tumors into “hot” ones, it is necessary not only to alter the phenotype of resident macrophages but also to inhibit the recruitment of suppressive myeloid cells and promote the infiltration of anti-tumor immune cells. In this process, polyphenolic compounds function as “microenvironment fine-tuners.” Polyphenols are secondary metabolites found in plants [68, 69, 74], primarily categorized into two main classes: phenolic acids and flavonoids. Common types include flavonoids, phenolic acids, and stilbenes. Foods rich in polyphenols are believed to help prevent chronic diseases and neurodegenerative disorders [16, 82]. Additionally, studies on melanoma mice have demonstrated that polyphenols can exert antitumor properties in an immune system-dependent manner. [35]. Within the TME, these compounds significantly reduce the accumulation of M2-like TAMs in the tumor core by regulating the CCL2/CCR2 axis or CXCL family chemokines. Concurrently, they downregulate the expression of immune checkpoints such as PD-L1, facilitating the transition of the TME from an “immune-excluded” to an “immune-inflamed” phenotype. The following section analyzes how these compounds synergistically enhance host anti-tumor immunity via multi-target effects.

#### Flavonoids

Isobavachalcone (IBC), first extracted from *Psoralea corylifolia L.* (PCL) in 1968, is defined as an isoprenylated chalcone. Studies using IBC in IL-4-induced M2-type RAW264.7 cells have shown that it can reduce the expression of CD206, along with a decrease in TGF-β, MRC1, and Arg1 expression. This indicates that IBC can effectively inhibit M2 polarization of macrophages [79].

Icariin (ICA), derived from *Herba Epimedii*, has been shown in in vitro experiments to inhibit the proliferation of pancreatic cancer. It suppresses the development of pancreatic cancer in mice by inhibiting tumor-infiltrating M2 macrophages and polymorphonuclear myeloid-derived suppressor cells (PMN-MDSC), evidenced by a significant decrease in the proportion of CD206-positive lymphocytes. In M2-type RAW264.7 cells, the use of ICA reduced the expression of MRC1 and Arg1, likely through the inhibition of the STAT6 signaling pathway [183].

Icariin (ICA) has also been shown to exert anti-bone metastasis effects in prostate cancer by inhibiting M2 polarization. Specifically, it does this by suppressing SPI1, which weakens the transcriptional expression of CCL5, thereby inhibiting the M2 polarization of macrophages [11].

Garcinone E (GE), derived from *mangosteen*, exhibits various biological activities, including anti-inflammatory effects and the induction of apoptosis in cancer cells [94, 157]. In studies on breast cancer metastasis, GE was used to induce polarization of RAW264.7 and THP-1 cells into M1 or M2 types. The results showed that GE promoted M1 polarization (with an increase in the proportion of CD86 +) while inhibiting M2 polarization (with a decrease in the proportion of CD206 +). In vivo experiments revealed that GE reduced the levels of STAT6 in tumor tissues by approximately twofold and decreased the phosphorylation of STAT6 by about fourfold, indicating that its effects are mediated through the regulation of the STAT signaling pathway [105].

Baohuoside I (BHS), derived from *Epimedium koreanum Nakai*, has been studied for its effects on M2 macrophages, which typically promote tumor invasion and drug resistance. In experiments examining the changes in extracellular vesicles after co-culturing untreated cells with macrophages, it was found that treatment with 2.5 μM BHS inhibited M2 polarization of macrophages, as evidenced by a decrease in CD206 expression. This effect is associated with CXCL1, suggesting that BHS may play a role in modulating the tumor microenvironment by affecting macrophage polarization (Shengqi [138, 139, 141]).

Baicalein, derived from *Scutellaria baicalensis Georgi*, is known for its potential to alleviate ulcerative colitis (Y. [68, 69, 74]), and inhibit ferroptosis, thereby mitigating damage from cerebral ischemia–reperfusion (Ming [68, 69, 74]). In the context of breast cancer, Baicalein has been shown to suppress M2 macrophage polarization, as indicated by the decreased expression of CD206, Arg1, TGF-β1, and IL-10. Additionally, it induces a shift in the phenotype and function of M2 macrophages towards M1 macrophages, evidenced by increased expression of CD86, IL-12, and TNF-α [180].

Quercetin is a subclass of flavonoids and a naturally occurring active compound found abundantly in tea and onions. It possesses various biological activities, including anticancer and immune-modulating effects [120]. In mouse models of liver cancer, quercetin has been shown to exert its anticancer effects by promoting M1 polarization (indicated by an increase in CD86 +) while inhibiting M2 polarization (evidenced by a decrease in the proportion of CD206 + and a reduction in TNF-α levels). Additionally, quercetin also downregulates PD-L1 expression, further contributing to its anticancer properties [151].

Flavonoids and their derivatives demonstrate a high degree of consistency in tumor immunomodulation, remodeling the TME by inhibiting the M2 immunosuppressive phenotype. Notably, GE, Baicalein, and Quercetin also promote an M1 pro-inflammatory state. The core mechanism centers on the blockade of STAT6 signal transduction (as seen with ICA and GE), which directly abrogates the transcription of M2-associated genes (e.g., Arg1, CD206) by reducing the phosphorylation of key transcription factors. Furthermore, specific components exhibit precise targeting capabilities: ICA inhibits prostate cancer bone metastasis via the SPI1/CCL5 axis, BHS modulates the CXCL1-associated microenvironment, and Quercetin potentiates anti-tumor immunity by downregulating the PD-L1 immune checkpoint. Collectively, these agents achieve precise reprogramming of macrophage function primarily by intervening in STAT family signaling pathways and associated chemokine axes.

#### Others

Osthole is a phenylpropanoid compound derived from *Cnidium monnieri (Fructus Cnidii)* and is an important member of the coumarin family. It has been shown to possess neuroprotective, anti-inflammatory, and antitumor pharmacological effects (S. [161, 163]). In an in situ mouse model of pancreatic cancer, treatment with Osthole resulted in a decrease in the ratio of CD206 + /CD11b + cells in the spleen and tumors. Additionally, in M2-type BMDMs, Osthole downregulated the expression of MRC1, Ccl22, and TGF-β. Furthermore, studies using RAW264.7 cells demonstrated that Osthole reduced IL-4-mediated STAT6 phosphorylation, downregulated C/EBPβ expression, and inhibited ERK1/2 phosphorylation [133, 135].

Matairesinol (MAT) is a lignan compound derived from *Podocarpus macrophyllus* (Thunb.) Sweet, known for its various effects, including insecticidal, antioxidant, and antitumor properties (Tong [174, 175, 177]). Treatment with MAT resulted in increased expression of CD86, CCR7, and TNF-α in M2 macrophages, while the expression of M2 markers such as IL-10, CD206, TGF-β, and TGM2 decreased. These findings suggest that MAT has the potential to repolarize M2 macrophages towards an M1 phenotype [10].

Caffeic acid (CA) is a phenolic acid widely found in various plants, fruits, and vegetables, such as coffee and olives, and is known for its immunomodulatory and neuroprotective effects [99]. In the context of breast cancer, CA has been shown to exert anticancer effects through the FOXO1/FIS pathway. Interestingly, CA promotes an increase in CD86 expression in splenocytes of mice. Furthermore, in breast cancer mouse models treated with CA, there was an observed increase in CD80 expression within the tumors, indicating that CA has the potential to enhance M1 polarization [155]。

Curcumin is a polyphenolic compound derived from the *Gingeraceae* and *Araceae families*, characterized by its diketone structure. It possesses the ability to influence various biological targets and has demonstrated activity against a range of diseases [61]. In studies focused on ovarian cancer, treatment with Curcumin at concentrations of 5, 10, and 20 μM in TAMs resulted in a decrease in the proportion of CD206 + cells, while there was no significant change in the proportion of CD86 + cells. Additionally, analysis of TAM polarization-related factors revealed a decrease in the mRNA expression of IL-10, TGF-β, CCL-23, and CXCR2, alongside an increase in the mRNA expression of IL-1, IL-12, and TNF-α (Xi [70, 71]).

Tannins are complex polyphenolic compounds widely distributed in nearly all plant-based foods and beverages, known for their antibacterial and antiviral properties [116], In a study involving mouse models of hepatocellular carcinoma, treatment with the tannin fraction from *Phyllanthus emblica* *L*. (PE-TF) demonstrated an increase in the expression of iNOS and CD68, while inhibiting the expression of CD163. mRNA analysis within the tumors revealed a significant increase in M1 markers, including iNOS, TNF-α, and IL-12p40, alongside a notable suppression of the M2 marker TGF-β. These findings indicate that PE-TF has the potential to promote M1 polarization of macrophages while inhibiting M2 polarization [9].

These compounds intervene in macrophage polarization primarily by finely regulating transcription factor phosphorylation and remodeling cytokine profiles. Osthole exhibits the most comprehensive mechanism; it potently blocks the transcription of M2-type genes via multi-target inhibition of the STAT6, C/EBPβ, and ERK1/2 signaling cascades. Conversely, CA opens a novel regulatory pathway, specifically enhancing the expression of M1-related factors through the FOXO1/FIS axis. Furthermore, although the precise pathways for Curcumin and tannins remain to be fully elucidated, they functionally demonstrate significant “phenotypic switching” characteristics. By fundamentally altering the balance between pro-inflammatory (IL-12, TNF-α) and anti-inflammatory (IL-10, TGF-β) factors within the tumor microenvironment, they convert the immunosuppressive state into an anti-tumor state. In summary, Osthole inhibits M2 polarization, while CA promotes M1 polarization. Additionally, MAT, Curcumin, and Tannins exhibit the potential to enhance M1 polarization of macrophages while suppressing M2 polarization.

### Alkaloids

While the aforementioned polyphenolic compounds primarily exert a fine-tuning effect on the tumor microenvironment by modulating oxidative phosphorylation and the chemokine axis, the alkaloids to be discussed hereafter demonstrate a profound capacity for signaling reprogramming. Alkaloids are organic compounds with a cyclic structure that contain one or more basic nitrogen atoms. They are widely distributed in nature and are naturally occurring secondary metabolites found in both plants and animals [106]. As one of the most biologically active classes of natural products, alkaloids possess the capacity to penetrate complex cellular signaling networks, precisely targeting core nodes such as JAK/STAT, MAPK, or NF-κB that dictate macrophage fate. This intervention rapidly induces a phenotypic reversal of pro-tumorigenic M2-type TAMs. By upregulating the secretion of iNOS and pro-inflammatory cytokines, these compounds transform the “immuno-cold” environment into a “hot” environment capable of efficiently triggering T-cell responses. In the following section, we provide a detailed analysis of the specific molecular mechanisms by which several alkaloids regulate TAM polarization.

9-Hydroxycanthin-6-one, derived from *Ailanthus altissima*, has been studied in the context of ovarian cancer. In experiments, THP-1 cells were stimulated with conditioned medium from ovarian cancer cells, and the expression of macrophage receptors (MR) and Trem2, as well as pro-cancer factors such as IL-10, VEGF, MMP-2, and MMP-9, was assessed. The results indicated that 9-Hydroxycanthin-6-one significantly inhibited the expression of these markers [50]

Stachydrine (STA), sourced from *Leonurus artemisia*, is found in plants like *Leonurus* and *Citrus aurantium*, as well as in various bacteria. It shows significant efficacy in treating cardiovascular diseases and exhibits anticancer activity [40]. In a mouse model of colorectal cancer liver metastasis, STA significantly inhibited tumor metastasis. However, in macrophage-depleted models, STA showed no therapeutic effect, indicating its anticancer action is macrophage-dependent. STA also inhibited the expression of CD206, ARG1, Fizz1, Mgl2, and TGF-β1 in macrophages induced by conditioned medium from colorectal cancer cells, correlating with the suppression of the JAK2/STAT3 signaling pathway [37].

Lobeline, primarily derived from *Lobelia inflata*, has been shown to reverse P-glycoprotein-dependent tumor resistance [85]. In a mouse model of colon cancer, genes associated with M1-like macrophages, such as IL-1b, TNF, CXCL9, IL12a, CD86, CD80, CCR7, and CXCL9, were upregulated in the Lobeline group. Conversely, M2-like macrophage-related genes, including TGFβ1, VEGFα, EGF, IL6, IL10, ARG1, and CCL22, were significantly downregulated. The M1 polarization pathway (IFN-γ-JAK-STAT1) was activated, while the M2 polarization pathway (IL-6-JAK-STAT3) was inhibited. Single-cell transcriptome sequencing indicated that Lobeline promotes the transcription and translation of SLURP1. In mice injected with Slurp1^−/−^ MC38 cells, tumor tissues showed a decrease in M1 cells and an increase in M2 cells, suggesting that SLURP1 is involved in Lobeline's regulation of macrophage polarization. Further studies confirmed that MAPK14, a transcriptional regulator involved in modulating multiple gene expressions, is a target protein of Lobeline, inhibiting SLURP1 transcription and translation through its interaction with p53 [179].

Dictamnine, derived from *Dictamnus dasycarpus Turcz.*, exerts anticancer effects in colorectal cancer by inhibiting the expression of M2-related markers such as ARG1, TGF-β, IL-10, and CD163. This action is associated with the MAPK signaling pathway [192].

Rutaecarpine (RUT), derived from *Euodia ruticarpa*, exhibits extensive pharmacological effects in treating various cardiovascular, cerebrovascular, and metabolic diseases [128]. Additionally, RUT significantly inhibits prostate cancer. In studies involving RUT-treated prostate cancer mouse models, factors secreted by peritoneal macrophages showed a significant increase in M1-related markers TNF-α, IL-1β, and IL-6, while M2 marker IL-10 remained unchanged. This suggests that RUT may enhance tumor immunity and exert its anticancer effects by promoting M1 polarization of macrophages [75].

Alkaloids exhibit a highly precise “polarization balance” regulation of the JAK/STAT signaling network. STA exerts macrophage-dependent anti-metastatic effects by inhibiting the JAK2/STAT3 pathway. In contrast, Lobeline demonstrates bidirectional regulatory characteristics: it activates the IFN-γ-JAK-STAT1 pathway to induce M1 polarization while simultaneously blocking the IL-6-JAK-STAT3 pathway to suppress M2 polarization. The underlying mechanism involves targeting the MAPK14 protein and mediating the transcription of SLURP1 via p53. Additionally, Dictamnine similarly attenuates M2-type features through the MAPK pathway. Collectively, these studies highlight the ability of alkaloid compounds to achieve precise reprogramming of TAMs from a pro-tumor to an anti-tumor state by intervening in intracellular kinase cascades. In alkaloids, 9-Hydroxycanthin-6-one, SAT, and dictamnine exert their effects by inhibiting M2 polarization, while RUT promotes M1 polarization. Lobeline, on the other hand, simultaneously enhances M1 polarization and inhibits M2 polarization to exert its anticancer effects.

### Polysaccharide

In the search for natural drivers capable of igniting cold tumors, polysaccharide components derived from traditional herbal medicines have demonstrated exceptional potential for innate immune activation. Polysaccharides are natural macromolecules typically composed of multiple monosaccharides linked by glycosidic bonds in either linear or branched forms. They are widely found in plants, microorganisms, algae, and animals. Similar to proteins and nucleic acids, polysaccharides are essential macromolecules in biological processes, playing crucial roles in intercellular communication, cell adhesion, and molecular recognition within the immune system [168]. Owing to their distinct molecular structural features, polysaccharides frequently function as prototypical agonists for Pattern Recognition Receptors (PRRs) [98], potently inducing the production of iNOS and NO in macrophages. This robust M1 polarization not only facilitates direct tumoricidal activity but also activates the downstream adaptive immune system by enhancing the antigen-presenting capacity of macrophages. This cascading response, propagating from localized activation to systemic engagement, constitutes a vital mechanism for maintaining long-term immunosurveillance within the TME and preventing immune evasion. This section summarizes several anticancer polysaccharides and their roles in remodeling macrophage functionality.

Lycium barbarum polysaccharides (LBP), derived from *Lycii fructus*, exert antioxidant and antitumor effects through various signaling pathways, including NF-κB, PI3K-Akt-mTOR, and Wnt-β-catenin [108]. In breast cancer mouse models, tumors from LBP-treated mice exhibited stronger CD86 fluorescence staining and weaker CD206 staining compared to untreated controls. Co-culture experiments with BMDM and 4T1 cells revealed increased expression of M1 markers such as TNF-α and IL-1β, indicating that LBP promotes M1 polarization of macrophages. This effect is associated with the activation of the TLR4-MyD88-IKK-NF-κB pathway, which enhances the innate immune response and repolarization of BMDM through upregulation of genes related to innate immunity, such as Trem1, Treml2, and Ly6a [76].

Apple polysaccharide (AP), derived from apple, alleviates colitis-associated colorectal cancer by regulating dysbiosis of the gut microbiota and activating the Wnt signaling pathway [73]. Additionally, AP has been found to activate the TLR4/NF-κB signaling pathway in macrophages, inducing M1 polarization, as evidenced by increased expression of iNOS, TNF-α, and IL-23, while suppressing the expression of Arg1, IL-4, and IL-10 [124].

Safflower polysaccharide (SPS), derived from Carthamus tinctorius L., exhibits antitumor and immunomodulatory effects. SPS-1, the primary active component of SPS, activates the NF-κB signaling pathway in macrophages, leading to the release of TNF-α, IL-6, and NO. Activated macrophages then induce apoptosis in colorectal cancer cells through this signaling pathway (Q. [136, 140]).

Arabinogalactan derived from *Pollen Typhae* is a potential immunomodulator [118]. In colorectal cancer, arabinogalactan (PTPS-1–2) activates macrophages through the NF-κB signaling pathway, leading to the release of TNF-α, IL-6, and NO. Analysis of M1 and M2 markers confirms an increase in the expression of iNOS and TNF-α genes, along with elevated ratios of CD86 and CD16. However, there are no significant changes in the M2 markers IL-4 and CD206 (Yongbin [158, 159]).

Astragalus polysaccharide (APS), derived from *Astragalus propinquus Schischk*, exhibits broad-spectrum antitumor effects and has potential for immunomodulation. In a liver cancer mouse model, the administration of APS significantly reduced the expression of CD206 and CD68 while increasing CD86 expression. Additionally, M1 polarization markers such as iNOS, TNF-α, and IL-1β showed increased gene expression levels, whereas the gene expression of Arg1 and IL-10 decreased (Chun [64, 65, 67]).

Huaier polysaccharides (HP), derived from *Trametes robiniophila Murr.*, may inhibit tumor growth through immunomodulation. HP treatment reduces the proportion of M2 macrophages (CD206) while promoting an increase in M1 macrophages (CD80) and related markers such as TNF-α, IL-9, and IL-1β. Analysis using 16S rRNA gene sequencing and untargeted metabolomics indicates that HP alters the gut microbiota and metabolites, potentially related to cholic acid (CDCA). Thus, HP suppresses hepatocellular carcinoma through gut microbiota-mediated M2 macrophage polarization (Xiaoxuan [70, 71]).

G. lucidum spore polysaccharide (GLSP), derived from *Ganoderma lucidum*, is known for its anti-inflammatory and immunomodulatory functions. When mouse peritoneal macrophage supernatant containing GLSP is added to liver cancer cells, it activates apoptosis-related pathways, inducing apoptosis in the cancer cells. In macrophages treated with GLSP, there is an increase in the expression of CD86, TNF-α, IL-1β, IL-6, and TGF-β1, while the proportion of CD206 decreases [121].

Among the aforementioned compounds, LBP, AP, and SPS primarily serve as ligands for the TLR4 receptor, driving macrophage polarization toward the M1 (pro-inflammatory/anti-tumor) phenotype via the canonical TLR4-MyD88-NF-κB signaling axis. Distinct from the previous groups of compounds, polysaccharides not only directly induce the release of iNOS and TNF-α through intracellular signaling cascades but also multi-dimensionally reshape the immune microenvironment via the gut microbiota-metabolism axis (e.g., Huaier polysaccharides regulating CDCA) or by upregulating innate immune receptors (e.g., Trem1). This regulation effectively suppresses the pro-tumorigenic effects of the M2 phenotype and, more crucially, directly induces tumor cell apoptosis by augmenting the cytotoxic factors of M1 macrophages, demonstrating the vast potential of polysaccharides in cancer immunotherapy. Notably, among polysaccharide-based natural products, LBP, AP, and APS have been found to exert their effects by bidirectionally regulating macrophage polarization through the NF-κB pathway, a regulatory capacity also shared by GLSP. Among polysaccharide natural products, LBP, AP, APS, and GLSP primarily exert their effects by bidirectionally regulating macrophage polarization through the NF-κB pathway, with PTPS-1–2 promoting M1 polarization and HP inhibiting M2 polarization.

### Saponins

If the aforementioned polysaccharides primarily 'ignite' cold tumors by mimicking PAMPs to leverage potent innate immune activation, then saponin compounds demonstrate a more profound capacity for epigenetic and transcriptional remodeling. Saponins are glycosides widely distributed in the plant kingdom, characterized by their structure, which includes a steroid or triterpenoid aglycone and one or more sugar chains [36]. As quintessential amphiphilic molecules among natural products, saponins not only engage in specific binding with membrane receptors such as TLR4 but also exhibit highly efficient regulation of core intracellular signaling pathways, including the STAT6, PPARG, and NF-κB axes. This multi-target intervention significantly inhibits the secretion of immunosuppressive factors (e.g., IL-10 and TGF-β) by M2 macrophages. Furthermore, by inducing lysosomal functional remodeling or interfering with chemokine release, saponins compel a phenotypic reversal in TAMs from a pro-tumorigenic to an anti-tumorigenic state. Through this profound phenotypic reprogramming, saponins are capable of reshaping the immunosuppressive "desert" into an "oasis" that supports T-cell infiltration and activation. This section explores the pivotal roles of several saponin-based natural products in remodeling the TME.

Anemoside A3 (A3), derived from *Pulsatilla chinensis*, shows potential in growth development and anti-inflammatory effects. In breast cancer, A3 promotes macrophage differentiation into M1-type via the TLR4/NF-κB/MAPK pathway, increasing M1 markers (CD86, IL-6, IL-1β) while decreasing CD206 and Arg1. This inhibits tumor growth and angiogenesis in the 4T1 metastatic model (P. [77, 80]). In breast cancer model, A3 treatment reduced CD86 and increased TLR4 expression, suggesting A3 activates TLR4 to enhance M1 polarization. Additionally, A3 upregulates TLR4/NF-κB/MAPK pathway proteins in macrophages and co-culture systems, with molecular docking indicating high binding affinity to the TLR4 complex. [165]

Astragaloside IV (AST), derived from *Radix Astragali*, inhibits M2-related markers (CD206, CCL4, PPARγ, Arg1, IL-10) in M2 macrophages without affecting M1 markers (CD86, iNOS, CCL5). Co-culture with tumor cells increases HMGB1 and TLR4 expression, which AST suppresses, thereby inhibiting tumor cell proliferation. This indicates that AST may block HMGB1-TLR4 signaling to inhibit IL-4/IL-13-induced M2 polarization (X. [136, 140]). In colorectal cancer, AST enhances the M1/M2 ratio by reducing extracellular vesicle release [184]. In hepatocellular carcinoma, AST activates the TLR4/NF-κB/STAT3 pathway, decreasing CD206 + cells and the mRNA levels of CD206 and TGF-β [93].

Ginsenoside RK3 (RK3), derived from *Panax ginseng*, reduces the expression of M2 macrophage markers (CD163, CD206, Arg1) in a glioblastoma mouse model, while CD80 and CD86 levels remain unchanged, indicating a decrease in M2 macrophage proportion in the tumor immune microenvironment. PPARG is identified as a potential target, with CCL2 facilitating TAM recruitment and positively correlating with PPARG. Thus, RK3 exerts its anticancer effects by inhibiting M2 macrophage polarization through the PPARG/CCL2 pathway [172, 182].

Tubeimoside-1 (TBMS1), a triterpenoid saponin derived from *Bolbostemmatis Rhizoma*, induces pyroptosis in colorectal cancer cells by targeting the PKM2/caspase-3/GSDME signaling axis. It increases the proportion of CD8 + T cells in mouse spleens and inhibits the activation of the STAT6 pathway in RAW 264.7 cells, thereby suppressing M2-like polarization (D. [44, 45]).

Saponins exhibit distinct receptor targeting and pathway specificity in the regulation of macrophage polarization. Pulsatilla saponin A3 directly binds to and activates the TLR4 receptor complex, triggering the NF-κB/MAPK cascade to bolster M1-type anti-tumor immunity. In contrast, while Astragaloside IV also involves the TLR4 pathway, its primary mechanism lies in the selective blockade of M2 polarization by inhibiting the HMGB1-TLR4 axis. Furthermore, Ginsenoside RK3 establishes a novel therapeutic route by intervening in M2 recruitment and polarization via the PPARG/CCL2 axis. Concurrently Tubeimoside I multi-dimensionally rewrites the tumor microenvironment by suppressing the canonical STAT6 pathway in conjunction with the induction of cancer cell pyroptosis. A3 promotes M1 polarization while inhibiting M2 polarization in macrophages. In contrast, AST, RK3, and TBMS1 exert their anticancer effects by suppressing M2 polarization.

### Steroids

Following the modulation of cell surface receptors and signaling axes by saponins, steroidal compounds—such as Bufalin and Arenobufagin—leverage their unique hydrophobic steroidal skeletons and superior membrane permeability to provide a more precise means of intervention for the immune remodeling of "cold" tumors. Steroids are a broad class of cyclopentane polycyclic hydrocarbons widely distributed in the biological world, also known as sterols or steroid compounds. In contrast to the broad-spectrum activation of the immune system by macromolecular polysaccharides, steroid-based constituents can directly penetrate the intracellular compartment to precisely downregulate M2-associated transcriptional programs. This is achieved by modulating key coactivators, such as SRC-3, or by interfering with the nuclear translocation of p50 NF-κB. Such regulation not only significantly reduces the release of inhibitory factors within the tumor stroma but also activates anti-tumor T-cell immunity by inducing M1-type polarization. Distinctively, these compounds can further "reinvigorate" the immunosuppressive microenvironment from a metabolic dimension by maintaining lipid metabolic homeostasis (e.g., via the PCSK9/LDL-R axis). The following sections will elucidate the mechanisms by which several steroid-based natural products modulate the polarization of TAMs.

Bufalin (BU) is a cardiac glycoside derived from *Bufo gargarizans*, classified as a bufadienolide. It is a C-24 steroid with various biological activities [17], Research indicates that BU exerts its anticancer effects in colorectal cancer by targeting SRC-3. In subcutaneous mouse models of colorectal cancer, BU enhances the antitumor efficacy of oxaliplatin (OXA) by inhibiting M2 polarization. Further studies in co-culture systems of macrophages and tumor cells reveal that BU does not directly inhibit M2 polarization in macrophages; instead, it targets chemotherapy-resistant cells to suppress M2 polarization. Specifically, BU modulates M2 macrophage polarization by targeting the SRC-3/MIF pathway in chemotherapy-resistant cells (J. [12, 14]). Additionally, BU can regulate M2 macrophage polarization by targeting the SRC-3/KLF4 pathway in highly metastatic colorectal cancer (mCRC) cells [81]. Also, BU can regulate M2 macrophage polarization by targeting the SRC-3/KLF4 pathway in highly metastatic colorectal cancer (mCRC) cells [134]. Clinically, colorectal cancer metastasis commonly occurs in the liver. In liver metastasis models, BU regulates M2 polarization of Kupffer cells (liver macrophages) through the SRC-3/IL-6 pathway (D. [126, 127]). In mouse models of hepatocellular carcinoma, significant changes in the expression of M1 and M2 genes are observed before and after BU treatment. M1-related genes such as IL-23, IL-12, TNF-α, IFN-γ, CD86, and Ccr7 show increased expression, while M2-related gene expression is suppressed. Further studies reveal that BU induces M1 polarization by reducing p50 NF-κB levels. Interestingly, BU drives M1 polarization through p50 expression to activate anti-hepatocellular carcinoma T cell immunity [169].

Arenobufagin (ARBU), also derived from *Bufo gargarizans*, belongs to the bufadienolide class due to differences in substituents on the parent nucleus. In co-culture systems of mouse peritoneal macrophages and liver cancer cells, ARBU induces M1 polarization in macrophages, evidenced by an increase in CD86 + cells and a decrease in CD206 + cells. Given the significant impact of lipid metabolism in the TME, the effects of ARBU on lipid metabolism in mouse models of hepatocellular carcinoma were assessed. Through screening with the KEGG database and validating differential gene expression via mRNA analysis, it was ultimately found that ARBU inhibits the PCSK9/LDL-R pathway in tumor cells, regulates cholesterol metabolism in the TME, and subsequently affect macrophage polarization [72]

This group of active constituents derived from toad venom demonstrates a sophisticated "cancer cell-macrophage" cross-talk regulatory mechanism. The BU is the steroid receptor coactivator SRC-3; it not only blocks the secretion of MIF or KLF4 in highly metastatic or drug-resistant cancer cells—thereby indirectly inhibiting M2 polarization—but also directly drives M1-type polarization via the p50 NF-κB pathway to activate T-cell immunity. Conversely, ARBU intervenes from a lipid metabolism perspective, modulating cholesterol homeostasis in the TME by targeting the PCSK9/LDL-R signaling axis to dictate macrophage fate. Such regulation, characterized by cellular metabolism and co-culture interactions, offers a novel immunopharmacological perspective for overcoming tumor drug resistance and suppressing metastasis.

### Others

Following a systematic discussion of classical constituents such as triterpenoids, alkaloids, and polysaccharides, the scope of this research has been further extended to include cannabinoids and lectins.

Cannabidiol (CBD) is a non-toxic cannabinoid that has been approved by the FDA for use in Epidiolex^®^ to treat Dravet syndrome, Lennox-Gastaut syndrome, and tuberous sclerosis complex, although its immunomodulatory effects are rarely reported. CBD restores the intrinsic antitumor activity of macrophages by inhibiting the PI3K-Akt signaling pathway and associated downstream target genes, shifting metabolic processes from oxidative phosphorylation and fatty acid oxidation to glycolysis. This results in increased iNOS expression and decreased Arg1 and CD206 expression in macrophages [123].

Korean mistletoe lectin (*Viscum album L. var. coloratum agglutinin*, VCA) has been shown to influence macrophage M1 and M2 polarization in breast cancer models at both 2D and 3D levels. This effect is characterized by an increase in M1 markers such as IL-6 and TNF-α, along with a decrease in M2 markers such as IL-10 and CCL18 [42].

These two classes of products exhibit distinct characteristics of metabolic remodeling and spatial structural response in macrophage regulation. The mechanism of CBD is particularly noteworthy; it intervenes in polarization not only by inhibiting the conventional PI3K-Akt signaling axis but also by "reprogramming" macrophages at the fundamental metabolic level. Specifically, it reverses macrophage function by shifting the metabolic mode from oxidative phosphorylation (conducive to M2 polarization) toward glycolysis (conducive to M1 polarization). Meanwhile, VCA demonstrates that natural products retain significant immunomodulatory potency even within the complex 3D tumor microenvironment, disrupting M2-dominant immunosuppression by remodeling the cytokine profile (upregulating TNF-α and downregulating IL-10).

## Prospects and limitations

**Fig. 3:**
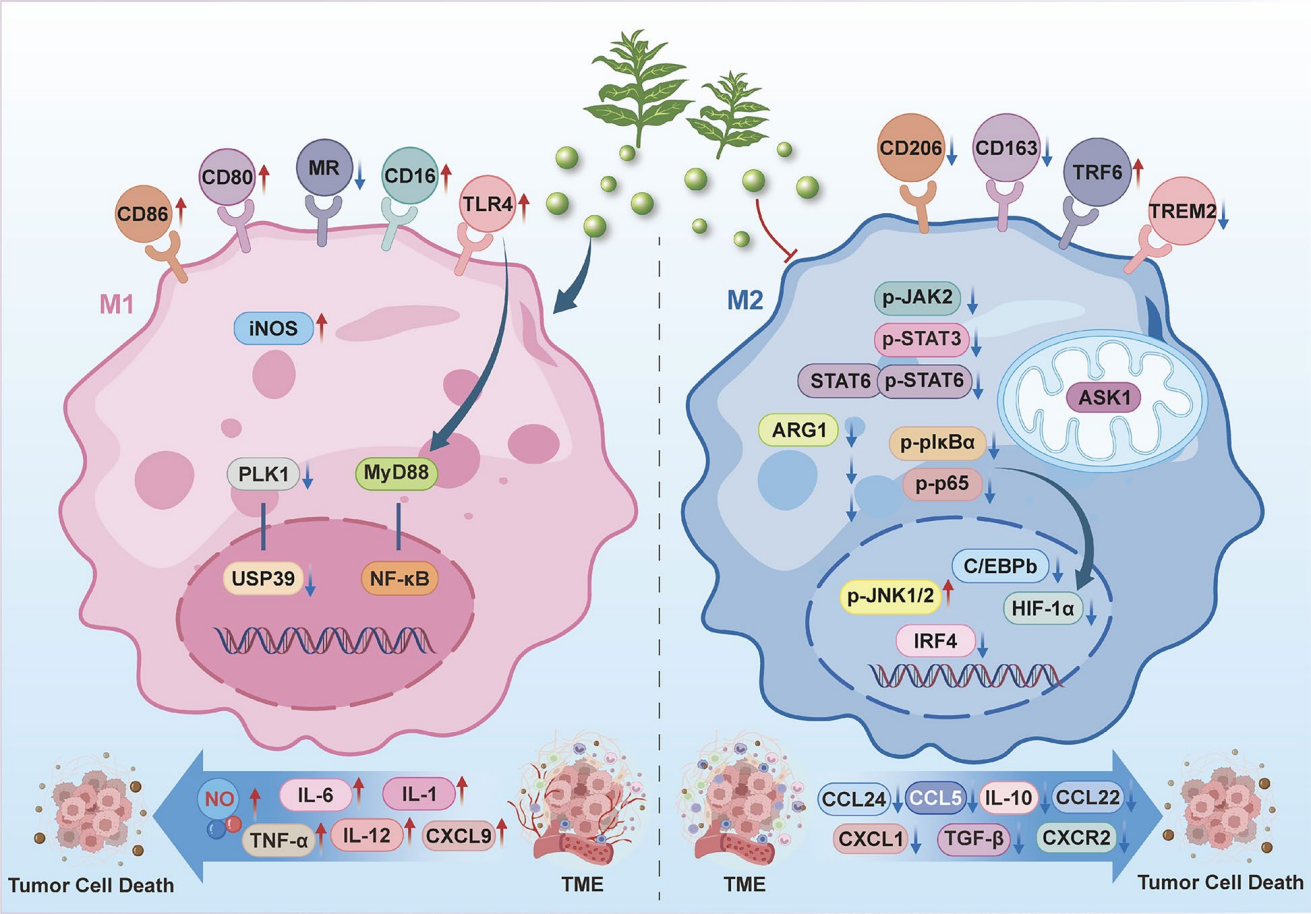
Mechanisms of natural product-induced macrophage polarization in cold tumors. (1) Natural products can induce M1 polarization of macrophages through the TLR4/MyD88/NF-κB and PLK1/USP39 signaling pathways, which is manifested by increased secretion of NO, IL-6, IL-1, TNF-α, IL-12, and CXCL9, alongside elevated expression of CD86, CD80, and CD16, and decreased expression of MR. (2) Natural products can affect TAM polarization by suppressing the expression of p-JAK2, p-STAT3, p-STAT6, p-IκBα/p-p65/HIF-1α, etc., which is manifested by decreased expression of CD206, CD163, ARG1, etc., representing a typical M2 polarization phenotype

As our understanding of tumors deepens, the TME has emerged as a breakthrough target for cancer treatment, with a close relationship existing between TAM and the TME [104, 110], Targeting TAM has the potential to alter the immunosuppressive state within tumors [25, 86, 152]. This article reviews the upstream factors that induce TAM M1 or M2 polarization and the impact of the cytokines released after polarization on the TME. It describes the relationship between TAM polarization and the immunosuppressive environment within the TME, as well as the crosstalk between TAM and other cells in the TME, such as T lymphocytes, CAFs, and TANs. The degree of TAM infiltration and the proportion of subpopulations in common cold tumors, such as breast cancer, hepatocellular carcinoma, and ovarian cancer, are significantly correlated with patient prognosis [92, 109, 170], Therefore, this review discusses natural products targeting macrophage polarization in cold tumors such as breast cancer, pancreatic cancer, and prostate cancer, along with their mechanisms of action **(**Fig. 3**),** and demonstrates their role in inhibiting tumor development.

### The advantages and potential of different natural products in regulating the anti-cold tumor effects of TAMs

Natural products are a rich repository of biologically active small molecules found in nature. These small molecules have the unique ability to rapidly and controllably modulate the activity of biological targets, which is why they have become active ingredients in many anti-tumor drugs [56]. In the reviewed natural products, polysaccharides such as LBP and AP can regulate TAM polarization through pathways like TLR4/NFκB, while most of the other compounds are primarily small molecules, typically used at concentrations below 100 μM. Notably, Toosendanin, garcinone E, and Bufalin exhibit cytotoxic effects on cancer cells at nanomolar concentrations (using models of prostate cancer, breast cancer, and colorectal cancer liver metastasis, respectively) and can also modulate macrophage polarization (promoting M1, enhancing M1 while inhibiting M2, and inhibiting M2). This indicates that these compounds possess high biological activity, suggesting that effective therapeutic doses can be lower, enhancing safety. One of the side effects of immunotherapy is the occurrence of cytokine storms [95]. Lower drug concentrations not only reduce cytotoxic effects and off-target effects but also potentially decrease the likelihood of cytokine storms. Additionally, the ability to exert effects at low concentrations suggests that these compounds may have high sensitivity to specific pathways related to macrophage polarization, such as those involving interferons (IFNs) or TLR signaling pathways [119]. This indicates their potential for precise modulation of polarization pathways.

Furthermore, among the natural products summarized in this article, Bufalin and Astragaloside IV are small molecules that have been frequently studied. They demonstrate macrophage reprogramming capabilities in colorectal cancer, hepatocellular carcinoma (both), and ovarian cancer (Astragaloside IV). Bufalin inhibits M2 polarization in colorectal cancer while simultaneously regulating both M1 and M2 in hepatocellular carcinoma. Astragaloside IV promotes M1 and inhibits M2 in colorectal cancer, and it also inhibits M2 polarization in ovarian cancer, as well as in models of colorectal cancer liver metastasis and hepatocellular carcinoma. This cross-cancer effectiveness highlights the broad-spectrum potential of natural products in modulating macrophage polarization across different cancer types, which may be closely related to the structures of these natural products. For instance, Bufalin binds to the Na⁺/K⁺-ATPase α subunit through its lactone ring and steroid nucleus, inducing conformational changes that trigger downstream responses. Additionally, it inhibits the SRC (steroid receptor coactivator, expressed in multiple cancer types) pathway, leading to downstream effects that regulate macrophage polarization. [13, 142]. Therefore, by analyzing the structures of these drugs, we can infer their key functional groups and targets. This information can serve as a reference for the development of derivatives with broad-spectrum anti-cancer effects and for the discovery of novel immunomodulators.

### Re-evaluation of the advantages and disadvantages of TAMs in anti-tumor therapy and improvement measures

From a regulatory perspective, natural products drive the transition from “cold” to “hot” tumors by targeting TAM polarization. This process represents more than a simple phenotypic switch; it constitutes a comprehensive immune remodeling of the TME. Once TAMs are reprogrammed into the M1 phenotype, they not only enhance direct phagocytosis and cytotoxicity against tumor cells but also dismantle the “immune desert” state by releasing pro-inflammatory cytokines and chemokines, such as TNF-α, IL-12, and CXCL9/10. The distinct advantage of natural products in this context lies in their poly-pharmacology. Compared to single-target synthetic drugs, natural products can simultaneously intervene across multiple dimensions, including signal transduction (e.g., JAK/STAT, NF-κB), metabolic reprogramming (e.g., glycolytic switching), and epigenetic modifications. This integrated multi-pathway regulation effectively overcomes tumor heterogeneity and reduces the risk of drug resistance arising from single-pathway blockade. However, elucidating the specific mechanisms underlying these poly-pharmacological effects remains challenging. Recent advancements in various detection technologies have provided new possibilities for clarifying these multi-target mechanisms [96, 113]. Furthermore, Natural products often exhibit multi-target effects, impacting not only intended targets but also other pathways, which may lead to adverse reactions. To mitigate these effects, researchers can enhance drug targeting and specificity by utilizing nanocarriers. Additionally, the immunosuppressive mechanisms within the TME vary among different cancer types; for instance, in hepatocellular carcinoma, macrophages accumulate, but the proportion of M2-related macrophages exceeds that of M1 [3], Therefore, it is essential to select drugs based on the specific conditions of the TME for each cancer type to enhance drug targeting.

### The potential of reshaping TAMs to enhance the immune microenvironment and synergize with ICI

Furthermore, existing immunotherapies, such as ICIs, often exhibit suboptimal therapeutic efficacy due to T-cell exhaustion, low PD-1 expression, and impaired antigen presentation (e.g., reduced MHC-I expression) [143]. Previous studies have demonstrated that combining ICIs with other treatments, such as chemotherapy, can synergistically circumvent tumor resistance to ICIs [131]. Cellular immunotherapies, including CAR-T cell therapy, face significant hurdles in solid tumors like prostate cancer, such as insufficient T-cell trafficking and an immunosuppressive TME [29]. The ability of natural products to remodel macrophage polarization and enhance immune infiltration provides a theoretical basis for achieving synergistic effects with these therapies. Consequently, combining natural products with ICIs (e.g., PD-1/PD-L1 inhibitors) or CAR-T therapy may cooperatively augment the immune response. However, considerations regarding pharmacological potency and the discordant pharmacokinetic profiles of these agents in vivo necessitate a careful evaluation of dosing schedules and timing.

### The challenge of targeting and regulating macrophage polarization with natural products

Although numerous natural products targeting macrophage polarization have been identified, several challenges persist: (1) Natural products often have low bioavailability and solubility, which limits their effectiveness in vivo. Given the ease of modification of small molecular structures, one approach to improve these shortcomings is to modify the molecular structure. For example, adding alanine or glutamic acid to the 7-O and/or 3-O positions of quercetin can create quercetin derivatives that enhance its properties [2, 4], (2) Many natural active small molecules exhibit significant anti-cancer effects; however, there is a lack of in-depth exploration of specific targets. Most articles in this review focus on the changes in classical pathways and corresponding cytokines related to TAM polarization after the action of natural products. Few studies investigate the direct targets of natural products and the specific targets that may regulate TAM polarization. The application of artificial intelligence to identify small molecule drugs from natural products has already been implemented in practice [173], Similarly, utilizing artificial intelligence to identify potential targets of small molecules is also feasible. Clarifying the mechanism of action and specific targets can optimize drug efficacy and safety. In the future, these small molecules could be combined with other drugs to inhibit off-target effects, thereby enhancing their therapeutic efficacy and safety, (3) Current research methods primarily focus on the effects of natural products on macrophages . To better simulate the in vivo environment, researchers should employ co-culture models of tumors and macrophages to investigate the mechanisms of action of natural products. In vivo experiments, due to the crosstalk among immune cells within the TME, researchers can also explore the effects of natural products on the interactions between immune cells to further clarify the specific actions of natural products on macrophages.

In summary, this review highlights natural products that target macrophage polarization in cold tumors, providing researchers with candidate drugs for cold tumor treatment. In future studies, researchers can design and construct more specific derivatives based on the structures of these natural products or develop more effective drug delivery systems to achieve targeted modulation of macrophages without affecting other cells. Additionally, exploring the direct targets of these drugs can further elucidate the mechanisms regulating macrophage polarization. Therefore, natural products hold significant potential as immune modulators that can convert tumors from “cold” to “hot,” particularly in the context of targeting macrophage polarization and immunotherapy.

## Supplementary Information

Supplementary Material 1

## Data Availability

No data was used for the research described in the article.

## Abbreviations

ARG1: Arginase-1
CAFs: Cancer-associated fibroblasts
CCR: Chemokine receptor
CTLA-4: Cytotoxic T lymphocyte-associated antigen 4
CXCL: Chemokine (C-X-C motif) ligand
DC: Dendritic cell
ECM: Extracellular matrix
Gal: Galectin-1
IL: Interleukin
ICIs: Immune checkpoint inhibitors
IFN: Interferon
iNOS: Inducible nitric oxide synthase
IRF1: Interferon regulatory factor 1
LPS: Lipopolysaccharide
M-csf: Macrophage colony-stimulating factor
MDSCs: Myeloid-derived suppressor cells
METTL3: Methyltransferase-like 3
MHC: Major histocompatibility complex
MMR: Mismatch match repair
MSI-H: High microsatellite instability colorectal cancer
NF-κB: Nuclear factor κB
NK: Natural killer
NPs: Natural products
PD-1: Programmed cell death protein 1
PD-L1: Programmed cell death ligand 1
PI3K: Phosphatidylinositol-3-Kinase
PPARG: Peroxisome proliferator activated receptor gamma
STAT3: Signal transducer and activator of transcription 3
TAMs: Tumor-associated macrophages
TAN: Tumor-associated neutrophils
TGF: Transforming growth factor
Th: T helper
TLRs: Toll like receptors
TME: Tumor microenvironment
Tregs: Regulatory T cells
TNF: Tumor necrosis factor
