# Supplementary figures and images for: Targeting tumor-associated macrophages through phytochemicals: a promising strategy for cold tumor therapy
Source: Chin Med. 2026 Apr 28;21:122. doi: 10.1186/s13020-026-01401-4 (PMC13122897; doi:10.1186/s13020-026-01401-4)

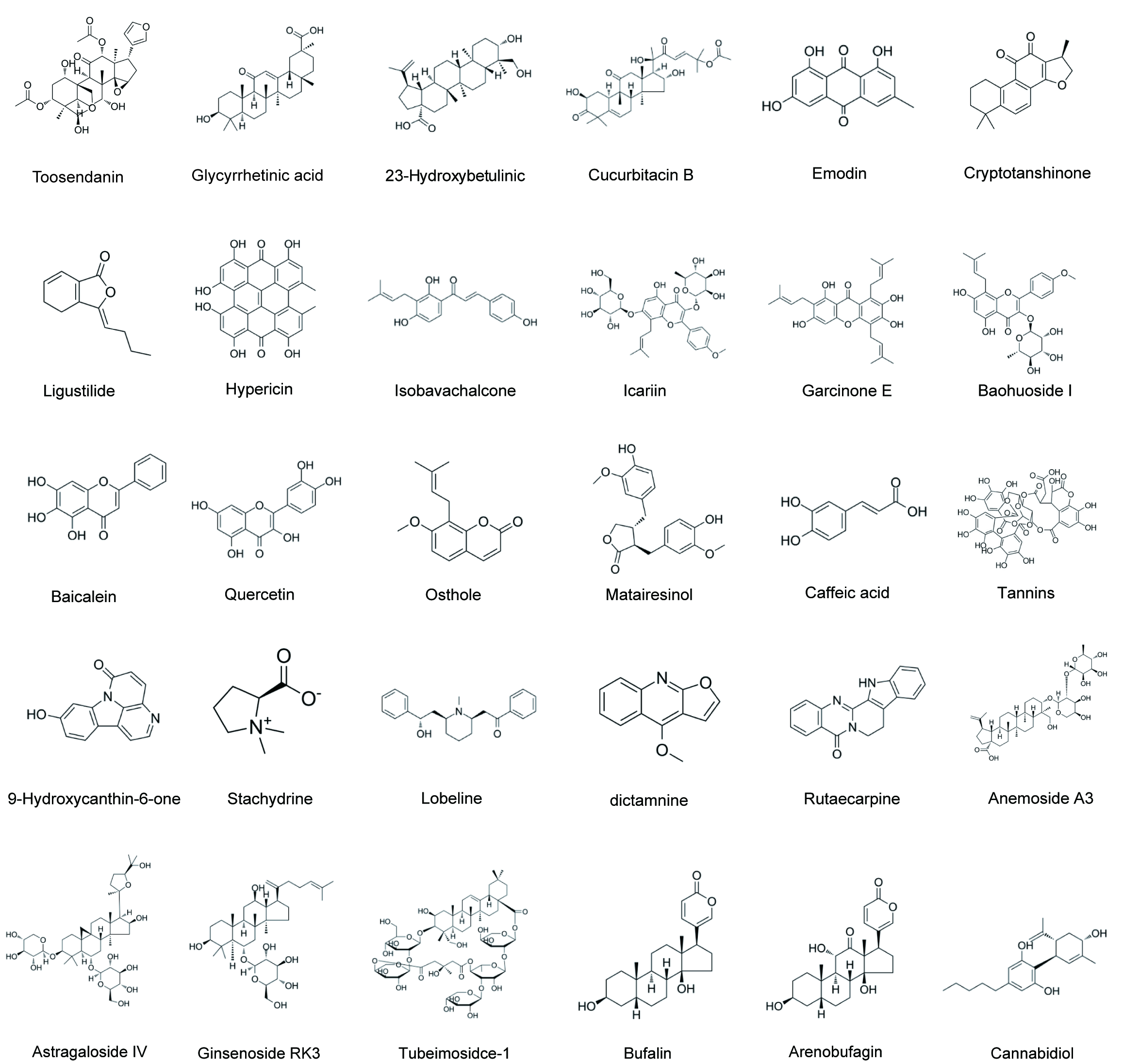

Supplement: Supplementary file 1 — Supplementary Material 1 [file 13020_2026_1401_MOESM1_ESM.tif]
